# Post-antibiotic era in hemodialysis? Two case reports of simultaneous colonization and bacteremia by multidrug-resistant bacteria

**DOI:** 10.1590/2175-8239-JBN-2020-0070

**Published:** 2020-09-11

**Authors:** Johanna M. Vanegas, Lorena Salazar-Ospina, Gustavo A. Roncancio, Julián Builes, Judy Natalia Jiménez

**Affiliations:** 1 Universidad de Antioquia, Escuela de Microbiología, Grupo de Investigación en Microbiología Básica y Aplicada, Medellín, Colombia.; 2 Clínica CardioVID, Departamento de Enfermedades Infecciosas, Medellín, Colombia.; 3 Hospital San Vicente Fundación, Departamento de Nefrología, Medellín, Colombia.

**Keywords:** Antimicrobial resistance, Carriage, Bacteremia, Resistência antimicrobiana, Transferência, Bacteremia

## Abstract

The emergence of resistance mechanisms not only limits the therapeutic options for common bacterial infections but also worsens the prognosis in patients who have conditions that increase the risk of bacterial infections. Thus, the effectiveness of important medical advances that seek to improve the quality of life of patients with chronic diseases is threatened. We report the simultaneous colonization and bacteremia by multidrug-resistant bacteria in two hemodialysis patients. The first patient was colonized by carbapenem- and colistin-resistant *Klebsiella pneumoniae*, carbapenem-resistant *Pseudomonas aeruginosa*, and methicillin-resistant *Staphylococcus aureus* (MRSA). The patient had a bacteremia by MRSA, and molecular typing methods confirmed the colonizing isolate was the same strain that caused infection. The second case is of a patient colonized by extended-spectrum beta-lactamases (ESBL)-producing *Escherichia coli* and carbapenem-resistant *Pseudomonas aeruginosa*. During the follow-up period, the patient presented three episodes of bacteremia, one of these caused by ESBL-producing *E. coli.* Molecular methods confirmed colonization by the same clone of ESBL-producing *E. coli* at two time points, but with a different genetic pattern to the strain isolated from the blood culture. Colonization by multidrug-resistant bacteria allows not only the spread of these microorganisms, but also increases the subsequent risk of infections with limited treatments options. In addition to infection control measures, it is important to establish policies for the prudent use of antibiotics in dialysis units.

## RESUMO

O surgimento de mecanismos de resistência não apenas limita as opções terapêuticas para infecções bacterianas comuns, mas também piora o prognóstico em indivíduos com condições que aumentam o risco de infecções bacterianas. Assim, a eficácia de importantes avanços médicos que buscam melhorar a qualidade de vida de pacientes com doenças crônicas está ameaçada. Relatamos a colonização e bacteremia simultâneas por bactérias multirresistentes em dois pacientes em hemodiálise. O primeiro paciente foi colonizado por Klebsiella pneumoniae resistente a carbapenem e colistina, Pseudomonas aeruginosa resistente a carbapenem e Staphylococcus aureus resistente a meticilina (MRSA). O paciente apresentou bacteremia por MRSA, e os métodos de tipagem molecular confirmaram que o isolado colonizador era a mesma cepa que estava causando infecção. O segundo caso é de um paciente colonizado por Escherichia coli produtora de beta-lactamases de espectro estendido (ESBL) e Pseudomonas aeruginosa resistente ao carbapenem. Durante o período de seguimento, o paciente apresentou três episódios de bacteremia, um deles causado por E. coli produtora de ESBL. Os métodos moleculares confirmaram a colonização pelo mesmo clone de E. coli produtora de ESBL em dois momentos, mas com um padrão genético diferente da cepa isolada da hemocultura. A colonização por bactérias multirresistentes aumenta o potencial não apenas da disseminação desses microrganismos, mas também do risco subsequente de infecções com opções limitadas de tratamentos. Além das medidas de controle de infecção, é importante estabelecer políticas para o uso prudente de antibióticos nas unidades de diálise.

## INTRODUCTION

Antimicrobial resistance has complicated the treatment of patients with bacterial infections by limiting the available options^1^_,_ 2. This situation has led the World Health Organization to warn of the arrival of a post-antibiotic era, in which common or previously easily-treated infections lead to therapeutic failures and deaths as a result of the simultaneous presence of different mechanisms of resistance^2^^,^ 3.

Patients with chronic renal failure on hemodialysis are highly susceptible to colonization and development of bacterial infections, with percentages exceeding those reported in individuals with other types of exposure to health care^4^. Bacterial infections are the second most common cause of hospitalization and death after cardiovascular disease and the risk of bacteremia is 26 times higher in comparison with the general population^4^. Likewise, the spread of resistant bacteria has been increasingly reported in this group of patients, who circulate continuously between the hospital environment and the community^5^^,^ 6. In this way, it has been described that 28% of these patients may be colonized by at least one resistant microorganism and that colonization generates a higher risk of infection, with a worse prognosis and with mortality rates up to 2.8 times higher compared to the general population^4^.

In this paper, we report the simultaneous colonization and bacteremia by multidrug-resistant bacteria in two hemodialysis patients included in a cohort study in which colonization by these microorganisms in stool, nostrils, and skin was evaluated at three time-points (at the beginning of the study, at month two and month six), in a renal unit of Medellín, Colombia. To refine the analysis, we used molecular typing methods to confirm if the patients were infected with the same multidrug-resistant strain that had been previously identified colonizing them.

The study protocol was approved by the Bioethics Committee for Human Research at the University of Antioquia (CBEIH-SIU) (approval no.18-35-819) and written informed consent was obtained from each subject.

## CASE PRESENTATION

### CASE 1

The first case is a 90-year-old man with a history of type II diabetes mellitus, arterial hypertension, and remission of colon adenocarcinoma. At the time of admission to the study, the patient had been on hemodialysis for four years and had a tunneled jugular dialysis catheter due to the dysfunction of different arteriovenous fistulas. As background, he reported hospitalization and antibiotic use (aztreonam) in the last six months. In addition, he complained of itching and frequent scratching around the insertion site of the catheter. The patient was positive in two of the three screenings for intestinal colonization by carbapenem-resistant *Klebsiella pneumoniae* and *Pseudomonas aeruginosa,* according to CLSI criteria. The *K. pneumoniae* isolate was positive for KPC carbapenemase by PCR and presented simultaneous resistance to colistin. Although the *mcr* plasmid gene that generates the transferable resistance to colistin was not detected, the alteration of the *mgrB* gene was mediated by the insertion sequence ISKpn25.

**Figure 1:**
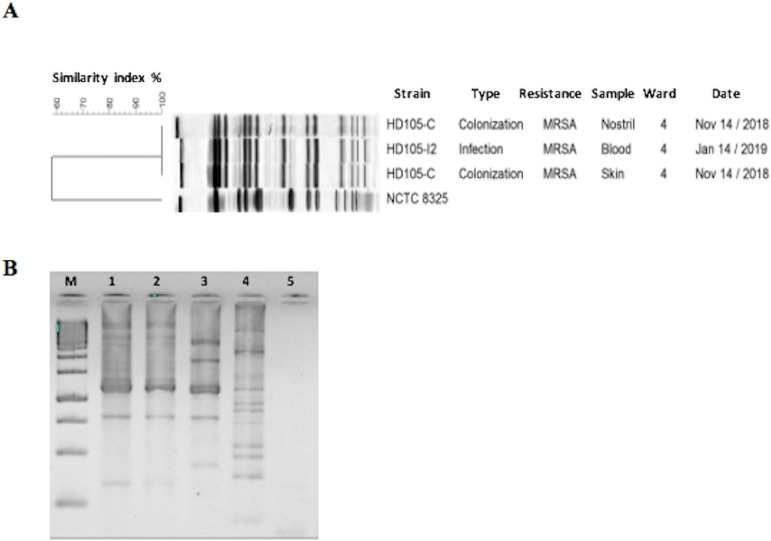
A. Pulsed-field gel electrophoresis (PFGE) with *Sma*I digestion. DNA fragment patterns were normalized using *S. aureus* strain NCTC8325. Cluster analysis was performed using the Dice coefficient in BioNumerics software version 6.0 (Applied Maths, Sint-Martens-Latem, Belgium). Dendrograms were generated by the unweighted pair group method using average linkages (UPGMA), with 1% tolerance and 0.5% optimization settings. B. Enterobacterial Repetitive Intergenic Consensus (ERIC). Samples: M (Marker), 1 (*E. coli* ESBL+, screening 1), 2 (*E. coli* ESBL+, screening 2), 3 (*E. coli* ESBL+, screening 3), 4 (isolate from blood), and 5 (negative control).

In the third screening, the patient was positive for MRSA in the nostrils and on the skin around the catheter insertion site. Two months later, he presented an episode of bacteremia due to this same bacteria. He received treatment with vancomycin and required dialysis catheter replacement. When processing the MRSA isolates from colonization and infection by pulsed-field gel electrophoresis (PFGE), it was confirmed that they corresponded to the same bacterial clone, which led to the conclusion that the colonizing strain was the same that caused the infection (Figure 1A). Laboratory markers of inflammation, malnutrition and renal function and echocardiogram results are shown in Table 1.

**Table 1:** Laboratory markers of inflammation, malnutrition, and renal function and echocardiogram results.

Marker or test	Case 1	Case 2
Albumin (g/dL)	4.20	2.99
Hemoglobin (g/dL)	12.2	10.2
C-reactive protein (mg/dL)	34.61	ND*
White blood cell count (cells/µL)	12700	3580
Blood urea nitrogen (mg/dL)	40	65
Potassium (mEq/L)	4.70	5.88
Calcium (mg/dL)	9.4	9.4
Chlorine (mEq/L)	105	ND*
Folic acid (ng/mL)	18.2	ND*
Vitamin B12 (pg/ml)	608	ND*
Creatinine (mg/dL)	3.69	ND*
Echocardiogram	Transesophageal echocardiogram without thrombi or evidence of endocarditis	Not performed

* No data.

### CASE 2

The second case is of a 57-year-old man with a history of systemic lupus erythematosus, arterial hypertension and primary hypothyroidism. The patient had lost a transplanted kidney and had been on hemodialysis four years by tunneled jugular catheter, due to a dysfunctional prior arteriovenous fistula caused by an aneurysm. The patient reported hospitalization and previous use of antibiotics (vancomycin and amikacin) in the last six months, as well as a history of multiple infections by multiresistant bacteria. He also reported itching and frequent scratching around the catheter insertion site. In all three screenings, the patient was positive for ESBL-producing *E. coli* and for carbapenem-resistant *Pseudomonas aeruginosa*. During the follow-up period, the patient presented three episodes of bacteremia caused by *Enterobacter cloacae*, and ESBL-producing *K. pneumoniae* and *E. coli*. The use of Enterobacterial Repetitive Intergenic Consensus (ERIC) confirmed colonization by the same clone of ESBL-producing *E. coli* in two of the three screenings, but with a different genetic pattern to the strain isolated from the blood culture (Figure 1B). Laboratory markers of inflammation, malnutrition, and renal function are shown in Table 1.

## DISCUSSION

The emergence of different resistance mechanisms threatens the success of important medical advances that improve the quality of life of patients with chronic diseases, and hemodialysis patients are no exception^4^. Colonization by MRSA is a known risk factor for the development of infections in hemodialysis patients. *Staphylococcus aureus* is the most frequently colonizing bacterium and MRSA colonization has been reported with a percentage ranging from 1.4 to 27% of hemodialysis patients, of which around 17 to 35% may develop bacteremia due to this same microorganism^4^. Likewise, a meta-analysis found that the risk of infections in patients colonized by this bacteria was more than 10 times greater compared to non-colonized patients (RR: 11.5; 95% CI, 4.7 to 28.0), with a 19% probability of developing infection in a period between 6 and 20 months in colonized patients compared to only 2% in non-colonized patients^7^. Persistent colonization by this microorganism worsens the prognosis of infections and is associated with a mortality rate increase of more than 85%^8^.

Unlike MRSA, few studies have evaluated colonization by multiresistant Gram-negative bacilli (MDR-GNB) in hemodialysis patients and their role in the development of infections^4^^-^ 6 (Table 2). This is worrisome, because the percentage of colonization by these microorganisms may be higher compared to MRSA colonization, as has been suggested by several authors^4^^-^ 6. The presence of ESBL generates resistance to penicillins, cephalosporins, and aztreonam, leaving carbapenems as the only treatment alternative^9^. Hemodialysis is an independent risk factor for infections by Gram-negative bacilli producing ESBL, so these patients have a higher risk of infection by these bacteria compared to susceptible isolates^9^. Even more worrying is the spread of carbapenemase-producing Gram-negative bacteria, because carbapenems, in addition to cephalosporins and other beta-lactams, are not effective against these microorganisms, leading to polymyxins such as colistin being the last treatment option^10^.

**Table 2:** Studies evaluating the colonization or infection by Gram-negative MDR bacilli in hemodialysis patients.

Author/year/country	Type of study	Colonization or infection	Bacteria	Resistance mechanism	Percentage
Bahramian A (15)	Descriptive	Infection	*K. pneumoniae*	NDM and ESBL	3/120 (2,5%)
2019					
Irán					
Rezende TFT (16)	Descriptive	Colonization	Gram negative bacilli	Carbapenemases	150/1092 (13.7%) 31 (2.8%)
2017					
Brasil					
Jamil B (17)	Descriptive	Bacteriemia	Gram negative bacilli	ESBL	17/46 (36.9%)
2016				Carbapenemase	7/46 (15.2%)
Pakistán				*P.aeruginosa* MDR	5/46 (10.9%)
Pop-Vicas A (5)	Descriptive	Colonization	Gram negative bacilli	Multidrug-resistant bacteria	11/67 (16.4%)
2008					
Estados Unidos					
Marchaim D (18)	Descriptive	Colonization	Gram negative bacilli	ESBL	9/105 (8.6%)
2005					
Israel					

The picture is complicated because many of the resistance mechanisms mentioned are in mobile genetic elements, which favors their rapid spread from one bacterium to another^11^. An example of this is colistin resistance, in which the insertion sequence ISKpn25 that alters the *mgrB* gene can be present in plasmids that also carry carbapenemases such as KPC, causing strains with simultaneous resistance to carbapenems and colistin, such as was observed in the case presented in this report^11^. Colistin resistance is of importance because it is one of the last treatment options for infections caused by carbapenem-producing bacteria^10^. Therefore, colonization by colistin-resistant microorganisms implies a potential risk of systemic infections with few treatment alternatives.

Because infections by multidrug-resistant bacteria are associated with a two to five-fold increase in morbidity and mortality compared to infections caused by susceptible isolates, the prevention of both colonization and infection by these microorganisms in patients in hemodialysis is crucial^10^. The screening of multidrug-resistant bacteria becomes more important in endemic countries, because the spread of these microorganisms exceeds the hospital environment and also occurs in outpatient services and in the community^12^. Therefore, prevention strategies should be focused on preventing the transmission of bacteria between patients, health care personnel and medical devices^4^. Because colonization is more frequent than infection and it can persist for long periods of time, the evaluation of prophylactic treatments in colonized patients is necessary to avoid the development of infections, oriented not only to nasal decolonization in the case MRSA, but other body sites, such as the catheter insertion site, where this and other resistant microorganisms can colonize^12^^,^ 13.

The vascular access type is also important to the development of bacteremia in hemodialysis patients. Of all access-related bloodstream infections, 70% occur in patients with catheters, so that the fistula is considered the preferred access due to lower infectious complications and lower cost^14^. However, in Colombia, as in other countries in Latin-America, most of hemodialysis patients have catheter and refuse to use fistula for fear or aesthetic reasons. Therefore, the effect of multidrug-resistant bacteria colonization on the development of infections such as bacteremia may be greater.

Finally, in addition to infection control measures, it is important to establish policies for the prudent use of antibiotics in dialysis units, because the use of these drugs is an important risk factor for the spread of drug-resistant bacteria. Given the few antibiotic treatment options, this is an urgent strategy that must be implemented.
